# Comparing hybrid and regular COVID-19 vaccine-induced immunity against the Omicron epidemic

**DOI:** 10.1038/s41541-022-00594-7

**Published:** 2022-12-15

**Authors:** Lei Huang, Francisco Tsz Tsun Lai, Vincent Ka Chun Yan, Franco Wing Tak Cheng, Ching Lung Cheung, Celine Sze Ling Chui, Xue Li, Eric Yuk Fai Wan, Carlos King Ho Wong, Ivan Fan Ngai Hung, Chak Sing Lau, Ian Chi Kei Wong, Esther Wai Yin Chan

**Affiliations:** 1 Centre for Safe Medication Practice and Research, Department of Pharmacology and Pharmacy, Li Ka Shing Faculty of Medicine, The University of Hong Kong, Hong Kong SAR, China; 2 Laboratory of Data Discovery for Health (D24H), Hong Kong Science Park, Hong Kong Science and Technology Park, Hong Kong SAR, China; 3 School of Nursing, Li Ka Shing Faculty of Medicine, The University of Hong Kong, Hong Kong SAR, China; 4 School of Public Health, Li Ka Shing Faculty of Medicine, The University of Hong Kong, Hong Kong SAR, China; 5 Department of Medicine, School of Clinical Medicine, Li Ka Shing Faculty of Medicine, The University of Hong Kong, Hong Kong SAR, China; 6 Department of Family Medicine and Primary Care, School of Clinical Medicine, Li Ka Shing Faculty of Medicine, The University of Hong Kong, Hong Kong SAR, China; 7 School of Pharmacy, University College London, London, UK; 8 Aston School of Pharmacy, Aston University, Birmingham, UK

**Keywords:** Epidemiology, Vaccines, Infectious diseases

## Abstract

Evidence on the effectiveness of COVID-19 vaccines among people who recovered from a previous SARS-CoV-2 infection is warranted to inform vaccination recommendations. Using the territory-wide public healthcare and vaccination records of over 2.5 million individuals in Hong Kong, we examined the potentially differential risk of SARS-CoV-2 infection, hospitalization, and mortality between those receiving two homologous doses of BNT162b2 or CoronaVac versus those with a previous infection receiving only one dose amid the Omicron epidemic. Results show a single dose after a SARS-CoV-2 infection is associated with a lower risk of infection (BNT162b2: adjusted incidence rate ratio [IRR] = 0.475, 95% CI: 0.410–0.550; CoronaVac: adjusted IRR = 0.397, 95% CI: 0.309–0.511) and no significant difference was detected in the risk of COVID-19-related hospitalization or mortality compared with a two-dose vaccination regimen. Findings support clinical recommendations that those with a previous infection could receive a single dose to gain at least similar protection as those who received two doses without a previous infection.

## Introduction

The Omicron variant of SARS-CoV-2 has rapidly emerged to dominate the global transmission since early November 2021^1^. Its high transmissibility and extensive immune escape impose substantial challenge to global health amidst the ongoing Coronavirus disease 2019 (COVID-19) pandemic^2,3^. As of June 30, 2022, over 300 million cases and one million deaths have been recorded worldwide since the Omicron variant was declared a variant of concern^4^, and the global weekly new COVID-19 cases had reached 4,182,217, among which more than 90% were caused by the Omicron variant^5^.

Both messenger RNA (mRNA) and inactivated COVID-19 vaccines are widely used as one of the most crucial public health measures to mitigate the impacts of the pandemic. Although the immune evasion of the Omicron variant hindered the vaccine effectiveness against infection^6^, evidence shows that COVID-19 vaccines can still effectively reduce the risk of severe conditions associated with Omicron infection^7^. Likewise, a prior SARS-CoV-2 infection can also confer immunity against reinfection among survivors^8–11^, while some studies suggest that the combination of a previous infection together with vaccination, i.e., hybrid immunity, might be more effective against reinfection and associated severe conditions compared to either vaccine-induced or natural immunity alone^12–14^. As the Omicron variant emerged, a high risk of breakthrough infection and reinfection was observed in vaccine recipients and those who were infected before^6,15^. More people may be in need of further vaccination against the virus, including those who were infected with previously circulating variants. However, the recommendation on vaccination for people with previous SARS-CoV-2 infection varies by region and is often unclear. For instance, France, Germany, and Italy recommend only one priming dose for those who recovered from a previous infection and with a healthy immune system^16^, while the United States recommend two priming doses regardless of infection history^17^. To date, whether a previous infection plus one priming dose of the vaccine can achieve a similar strength of protection as the typical two-dose vaccine regimen remains unclear, especially against the Omicron variant. Individuals with a previous infection are largely left confused as to whether further vaccination is favorable or necessary, although its relatively high safety has been supported by previous research^18^. Real-world evidence on the effectiveness of COVID-19 vaccines among people who recovered from a previous SARS-CoV-2 infection is much warranted to inform vaccination policies and uptake as well as infection control measures.

With 7.5 million population, Hong Kong has been implementing a dynamic zero-COVID policy since the virus started to spread in the community in January 2020^19^. The infection rate was largely kept under control in the previous four epidemic waves with daily new cases not exceeding 200 until the Omicron variant arrived in January 2022^20,21^. As of June 30, a total of 763,551 laboratory confirmed cases and 469,615 self-reported cases using rapid antigen tests (RAT) were documented during the fifth wave^22^. This surge overwhelmed the city’s healthcare system and the mortality rate increased noticeably, with over 9000 COVID-19-related deaths recorded during the fifth wave^20,22^. The Hong Kong government has been promoting vaccine uptake to reduce severe conditions associated with infection and to alleviate the pressure on the local healthcare system. The government recommend people infected before to receive one priming dose instead of both doses to be considered as fully vaccinated^23^. Using population-based vaccination records linked with a territory-wide public healthcare database in Hong Kong, this study examined the potentially differential risk of SARS-CoV-2 infection, COVID-19-related hospitalization, and COVID-19-related mortality between individuals receiving the regular two-dose regimen of BNT162b2 (Pfizer-BioNTech) or CoronaVac (Sinovac) versus those with a previous infection receiving only one dose amid the Omicron outbreak.

## Results

### Cohort characteristics

**Fig. 1:**
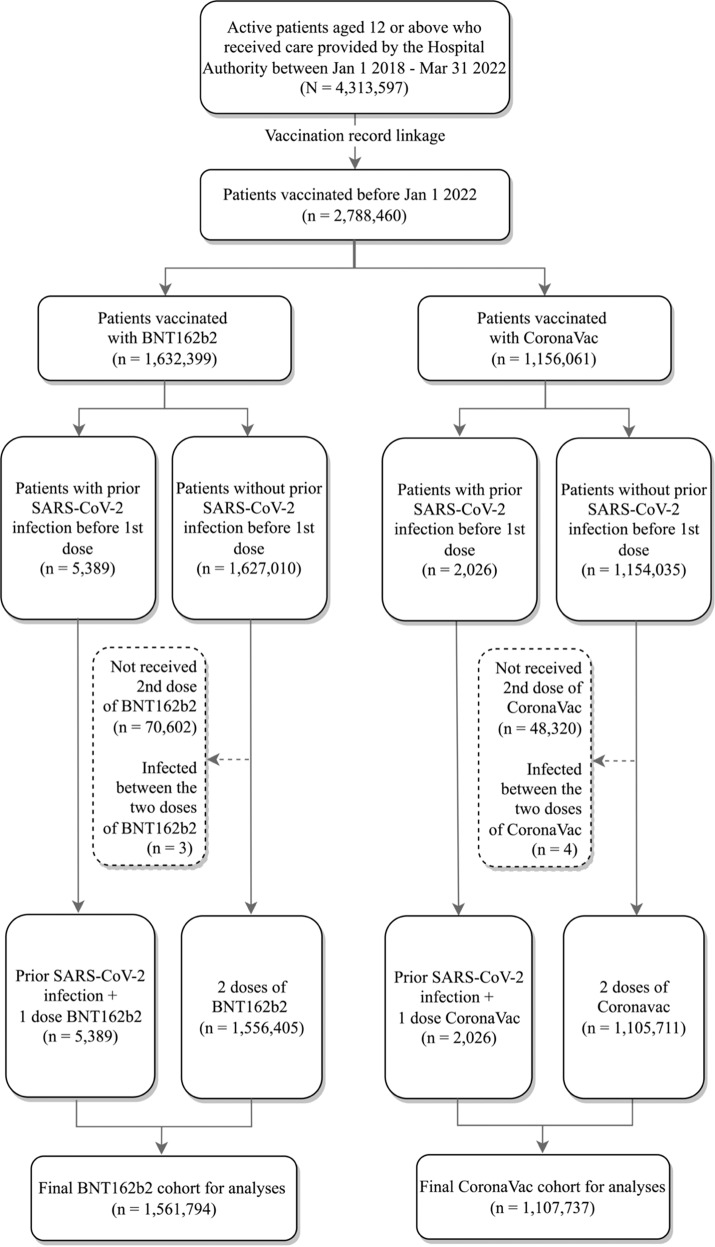
Cohort selection process. Boxes with dotted borders represent excluded participants.

In this study, vaccinated with previous infection refers to those who were infected with SARS-CoV-2 and then vaccinated with a single dose of the COVID-19 vaccine. The final cohort included 2,669,531 vaccine recipients, of which 7415 had prior SARS‑CoV‑2 infection (infection plus one dose of BNT162b2: 5389; infection plus one dose of CoronaVac: 2026), and 2,662,116 had no history of SARS‑CoV‑2 infection (two doses of BNT162b2: 1,556,405; two doses of CoronaVac: 1,105,711). Figure 1 shows the cohort selection process. The cohort characteristics are presented in Table 1. Among BNT162b2 recipients, the proportion of males was higher in the group with prior infection (49.1%) than that in group without prior infection (44.9%), and the mean age (in year) was younger in the group without prior infection (Mean = 45.85, Standard deviation [SD] = 17.04) compared to the group with prior infection (Mean = 44.28, SD = 16.40). Among CoronaVac recipients, sex was similar between the two groups while the mean age was younger in the group without prior infection (Mean = 56.86, SD = 14.40) compared to the group with prior infection (Mean = 54.93, SD = 14.56). For both vaccine types, the group with prior infection had longer gap between index date and the Omicron outbreak compared to the group with no prior infection. The history of most chronic conditions was similar between the two groups, though the proportion of people taking medication in the past 90 days was lower in the group with prior SARS‑CoV‑2 infection. Among individuals with a prior infection, the median interval between infection and vaccination was 234 days (Interquartile range [IQR] = 181) for BNT162b2 recipients and 258 days (IQR = 135) for CoronaVac recipients.

**Table 1:** Baseline characteristics of two-dose vaccine recipients without prior infection and one-dose recipients with prior infection.

	BNT162b2			CoronaVac		
	With prior SARS‑CoV‑2 infection	Without prior SARS‑CoV‑2 infection		With prior SARS‑CoV‑2 infection	Without prior SARS‑CoV‑2 infection	
*n*	5389	1,556,405	SMD^b^	2026	1,105,711	SMD^b^
Sex = Male (%)	2645 (49.1)	698,876 (44.9)	0.042	921 (45.5)	504,875 (45.7)	0.002
Age [mean (SD)]	44.28 (16.40)	45.85 (17.04)	0.094	54.93 (14.56)	56.86 (14.40)	0.133
Index date to outbreak^a^ [in days, mean (SD)]	176.42 (58.12)	153.71 (56.97)	0.395	174.38 (71.64)	161.28 (67.55)	0.188
Chronic conditions (%)						
Alcohol misuse	5 (0.1)	2842 (0.2)	0.001	5 (0.2)	2893 (0.3)	0.001
Asthma	42 (0.8)	14,287 (0.9)	0.001	21 (1.0)	10,028 (0.9)	0.001
Atrial fibrillation	23 (0.4)	5931 (0.4)	<0.001	17 (0.8)	8712 (0.8)	<0.001
Cancer lymphoma	4 (0.1)	993 (0.1)	<0.001	1 (0.0)	713 (0.1)	0.001
Cancer metastatic	2 (0.0)	2238 (0.1)	0.001	4 (0.2)	2167 (0.2)	<0.001
Cancer non-metastatic	16 (0.3)	9512 (0.6)	0.003	14 (0.7)	9206 (0.8)	0.001
Chronic heart failure	5 (0.1)	2787 (0.2)	0.001	11 (0.5)	4136 (0.4)	0.001
Chronic kidney disease	43 (0.8)	16,300 (1.0)	0.002	44 (2.2)	19,748 (1.8)	0.004
Chronic pain	90 (1.7)	27,762 (1.8)	0.001	35 (1.7)	27,425 (2.5)	0.008
Chronic pulmonary disease	18 (0.3)	4716 (0.3)	<0.001	13 (0.6)	7302 (0.7)	0.001
Chronic viral hepatitis B	38 (0.7)	14,024 (0.9)	0.002	28 (1.4)	17,010 (1.5)	0.001
Cirrhosis	2 (0.0)	1305 (0.1)	0.001	0 (0.0)	1410 (0.1)	0.001
Dementia	1 (0.0)	730 (0.0)	<0.001	2 (0.1)	1776 (0.2)	0.001
Depression	69 (1.3)	26,534 (1.7)	0.004	32 (1.6)	19,403 (1.8)	0.002
Diabetes	390 (7.2)	108,130 (6.9)	0.003	235 (11.6)	142,314 (12.9)	0.013
Epilepsy	8 (0.1)	2385 (0.2)	0.001	6 (0.3)	1846 (0.2)	0.001
Hypertension	678 (12.6)	213,350 (13.7)	0.011	401 (19.8)	274,336 (24.8)	0.050
Hypothyroidism	51 (0.9)	18,447 (1.2)	0.003	25 (1.2)	17,690 (1.6)	0.004
Inflammatory bowel disease	4 (0.1)	1137 (0.1)	<0.001	1 (0.0)	601 (0.1)	0.001
Irritable bowel syndrome	2 (0.0)	1636 (0.1)	0.001	4 (0.2)	1102 (0.1)	0.001
Multiple sclerosis	3 (0.1)	503 (0.0)	0.001	1 (0.0)	192 (0.0)	<0.001
Myocardial infarction	11 (0.2)	2328 (0.1)	0.001	10 (0.5)	3045 (0.3)	0.002
Parkinson’s disease	1 (0.0)	954 (0.1)	0.001	1 (0.0)	1235 (0.1)	0.001
Peptic ulcer disease	10 (0.2)	3673 (0.2)	<0.001	6 (0.3)	4914 (0.4)	0.001
Peripheral vascular disease	1 (0.0)	555 (0.0)	<0.001	1 (0.0)	789 (0.1)	0.001
Psoriasis	4 (0.1)	1869 (0.1)	<0.001	4 (0.2)	1500 (0.1)	0.001
Rheumatoid arthritis	8 (0.1)	3839 (0.2)	0.001	4 (0.2)	3255 (0.3)	0.001
Schizophrenia	10 (0.2)	3776 (0.2)	<0.001	7 (0.3)	4030 (0.4)	0.001
Severe constipation	81 (1.5)	29,601 (1.9)	0.004	45 (2.2)	33,280 (3.0)	0.008
Stroke or transient ischemic attack	22 (0.4)	9076 (0.6)	0.002	19 (0.9)	13,423 (1.2)	0.003
Medications (%)						
Renin–angiotensin system agents	361 (6.7)	124,195 (8.0)	0.013	227 (11.2)	153,502 (13.9)	0.027
Beta blockers	207 (3.8)	74,256 (4.8)	0.010	118 (5.8)	90,726 (8.2)	0.024
Calcium channel blockers	537 (10.0)	181,243 (11.6)	0.016	320 (15.8)	234,242 (21.2)	0.054
Diuretics	37 (0.7)	18,489 (1.2)	0.005	35 (1.7)	24,038 (2.2)	0.005
Nitrates	47 (0.9)	13,950 (0.9)	<0.001	35 (1.7)	18,675 (1.7)	<0.001
Lipid-lowering agents	502 (9.3)	181,597 (11.7)	0.024	293 (14.5)	224,652 (20.3)	0.058
Insulins	48 (0.9)	11,974 (0.8)	0.001	40 (2.0)	13,628 (1.2)	0.008
Antidiabetic drugs	319 (5.9)	94,392 (6.1)	0.002	194 (9.6)	121,405 (11.0)	0.014
Antiarrhythmic drugs	0 (0.0)	1053 (0.1)	0.001	1 (0.0)	1073 (0.1)	0.001
Oral anticoagulants	19 (0.4)	5319 (0.3)	0.001	10 (0.5)	7112 (0.6)	0.001
Antiplatelets	163 (3.0)	55,704 (3.6)	0.006	116 (5.7)	74,232 (6.7)	0.010
Steroid	40 (0.7)	8535 (0.5)	0.002	19 (0.9)	6730 (0.6)	0.003
Antidepressant	113 (2.1)	49,718 (3.2)	0.011	57 (2.8)	43,279 (3.9)	0.011
Antiviral drugs	82 (1.5)	16,212 (1.0)	0.005	59 (2.9)	15,792 (1.4)	0.015
Antibacterial drugs	154 (2.9)	46,977 (3.0)	0.001	66 (3.3)	35,050 (3.2)	0.001
Immunosuppressants	21 (0.4)	5161 (0.3)	0.001	18 (0.9)	3365 (0.3)	0.006

^a^Refers to the Omicron outbreak in Hong Kong starting from Jan 1, 2022.

^b^Standardized mean difference (SMD) for continues variables; proportion difference for categorical variables.

### Comparing hybrid and vaccine-induced immunity

Within the observation period, 127,085 (8.137%) SARS‑CoV‑2 infections were observed among BNT162b2 recipients and 92,961 (8.392%) SARS‑CoV‑2 infections were observed among CoronaVac recipients. The incidence rate per 100,000 person-day in the BNT162b2 group was 15.38 among individuals with previous infection and 37.21 among individuals without previous infection. The incidence rate per 100,000 person-day in the CoronaVac group was 15.89 among individuals with previous infection and 40.16 among individuals without previous infection. The estimated incidence rate ratios (IRR) and the corresponding 95% confidence intervals (CI) are presented in Table 2. Vaccine recipients infected in previous waves had a lower risk of SARS‑CoV‑2 infection during the Omicron outbreak regardless of vaccine type (BNT162b2: adjusted IRR = 0.475, 95% CI: 0.410, 0.550; CoronaVac: adjusted IRR = 0.397, 95% CI: 0.309, 0.511). Among BNT162b2 recipients, four people (0.074%) with prior SARS‑CoV‑2 infection and 3,343 people (0.215%) without prior SARS‑CoV‑2 infection experienced COVID-19-related hospitalization. Among CoronaVac recipients, two people (0.099%) with prior SARS‑CoV‑2 infection and 3,026 people (0.274%) without prior SARS‑CoV‑2 infection experienced COVID-19-related hospitalization. No significant difference in risk of COVID-19-related hospitalization was observed between vaccine recipients with and without prior SARS‑CoV‑2 infection for either BNT162b2 recipients (adjusted IRR = 0.394, 95% CI: 0.148, 1.406) or CoronaVac recipients (adjusted IRR = 0.433, 95% CI: 0.108, 1.733). No COVID-19-related mortality was observed in the group with prior SARS‑CoV‑2 infection for both vaccine types. Similar results were also observed in a series of sensitivity analysis (Supplementary Tables 1–4). No significant association between prior SARS‑CoV‑2 infection and the risk of COVID-19-related hospitalization was detected among vaccine recipients with the index date postponed by 14 days or COVID-19-related hospitalization defined by SARS‑CoV‑2 polymerase chain reaction (PCR) positive within 14 or 42 days before admission. When we included self-reported infection cases using RAT, the results were also similar to those of the main analysis, except that individuals who received a single dose of BNT162b2 after a previous infection had a lower risk of COVID-19 related hospitalization than individuals who received two doses of BNT162b2 with no previous infection, although the significance was marginal (adjusted IRR = 0.371, 95% CI: 0.140, 0.985).

**Table 2:** Incidence rate ratios of study outcomes between two-dose vaccine recipients without prior infection and one-dose recipients with prior infection.

	Number of individuals	Event	Total duration of follow-up (person-days)	Incidence rate^a^	Incidence rate ratio (95% confidence interval)^b^, *p* value	
					Crude	Adjusted^c^
*SARS-CoV-2 infection*						
BNT162b2						
Without prior SARS‑CoV‑2 infection	1,556,405	126,907	341,094,574	37.206	Ref	Ref
With prior SARS‑CoV‑2 infection	5389	178	1,157,210	15.382	0.413 (0.357, 0.479), <0.001	0.475 (0.410, 0.550), <0.001
CoronaVac						
Without prior SARS‑CoV‑2 infection	1,105,711	92,900	231,341,350	40.157	Ref	Ref
With prior SARS‑CoV‑2 infection	2026	61	383,863	15.891	0.396 (0.308, 0.509), <0.001	0.397 (0.309, 0.511), <0.001
*COVID-19 hospitalization*						
BNT162b2						
Without prior SARS‑CoV‑2 infection	1,556,405	3343	344,734,316	0.970	Ref	Ref
With prior SARS‑CoV‑2 infection	5389	4	1,162,207	0.344	0.355 (0.134, 0.934), 0.038	0.394 (0.148, 1.046), 0.062
CoronaVac						
Without prior SARS‑CoV‑2 infection	1,105,711	3026	233,887,234	1.294	Ref	Ref
With prior SARS‑CoV‑2 infection	2026	2	385,545	0.519	0.401 (0.100, 1.601), 0.196	0.433 (0.108, 1.733), 0.237
*COVID-19 mortality*						
BNT162b2						
Without prior SARS‑CoV‑2 infection	1,556,405	94	344,887,368	0.027	Ref	Ref
With prior SARS‑CoV‑2 infection	5389	0	1,162,367	0.000	–	–
CoronaVac						
Without prior SARS‑CoV‑2 infection	1,105,711	350	233,980,242	0.150	Ref	Ref
With prior SARS‑CoV‑2 infection	2026	0	385,579	0.000	–	–

^a^Incidence rate per 100,000 person-day.

^b^Incidence rate ratios for outcomes with zero events were not estimated.

^c^Adjusted for age, sex, number of days from the index date to the local Omicron outbreak (January 1, 2022), clinical history of chronic conditions before the index date (Supplementary Table 5), and medications prescribed within 90 days before the index date (Supplementary Table 6).

### Comparing natural and vaccine-induced immunity

Given that no differential risk of COVID-19-related hospitalization or mortality was detected between groups in the main analysis, we conducted a series of extended analyses to compare the risk between people infected before but with no subsequent COVID-19 vaccination and fully vaccinated people received two doses of BNT162b2 or CoronaVac with no prior infection. The results are presented in Table 3. Within the observation period, 64 SARS‑CoV‑2 infection (0.627%), six COVID-19-related hospitalization (0.059%) and one COVID-19-related death (0.010%) were observed among non-vaccine recipients infected in previous waves. Lower risk of infection during the Omicron outbreak was observed in those previously infected and unvaccinated, compared to those fully vaccinated without previous infection (BNT162b2: adjusted IRR = 0.622, 95% CI: 0.486, 0.795; CoronaVac: adjusted IRR = 0.746, 95% CI: 0.584, 0.953). No significant difference in risk of COVID-19-related hospitalization (BNT162b2: adjusted IRR = 0.793, 95% CI: 0.350, 1.799; CoronaVac: adjusted IRR = 2.197, 95% CI: 0.979, 4.932) or mortality (BNT162b2: adjusted IRR = 1.413, 95% CI: 0.151, 13.186; CoronaVac: adjusted IRR = 2.397, 95% CI: 0.317, 18.104) was detected between vaccine recipients and non-vaccine recipients infected in previous waves.

**Table 3:** Incidence rate ratios of study outcomes between vaccine recipients not previously infected and non-vaccine recipients with a previous infection.

	Number of individuals	Event	Total duration of follow-up (person-days)	Incidence rate^a^	Incidence rate ratio (95% confidence interval)^b^, *p*-value	
					Crude	Adjusted^c^
*SARS-CoV-2 infection*						
BNT162b2						
Vaccinated with 2 doses of BNT162b2	1,556,405	126,907	341,094,574	37.206	Ref	Ref
With history of infection and unvaccinated	10,207	64	3,299,894	1.939	0.052 (0.041, 0.067), <0.001	0.622 (0.486, 0.795), <0.001
CoronaVac						
Vaccinated with 2 doses of CoronaVac	1,105,711	92,900	231,341,350	40.157	Ref	Ref
With history of infection and unvaccinated	10,207	64	3,299,894	1.939	0.048 (0.038, 0.062), <0.001	0.746 (0.584, 0.953), 0.019
*COVID-19 hospitalization*						
BNT162b2						
Vaccinated with 2 doses of BNT162b2	1,556,405	3,343	344,734,316	0.970	Ref	Ref
With history of infection and unvaccinated	10,207	6	3,301,470	0.182	0.187 (0.084, 0.417), <0.001	0.793 (0.350, 1.799), 0.579
CoronaVac						
Vaccinated with 2 doses of CoronaVac	1,105,711	3,026	233,887,234	1.294	Ref	Ref
With history of infection and unvaccinated	10,207	6	3,301,470	0.182	0.140 (0.063, 0.313), <0.001	2.197 (0.979, 4.932), 0.056
*COVID-19 mortality*						
BNT162b2						
Vaccinated with 2 doses of BNT162b2	1,556,405	94	344,887,368	0.027	Ref	Ref
With history of infection and unvaccinated	10,207	1	3,301,618	0.030	1.111 (0.155, 7.971), 0.916	1.413 (0.151, 13.186), 0.762
CoronaVac						
Vaccinated with 2 doses of CoronaVac	1,105,711	350	233,980,242	0.150	Ref	Ref
With history of infection and unvaccinated	10,207	1	3,301,618	0.030	0.202 (0.029, 1.431), 0.109	2.397 (0.317, 18.104), 0.397

^a^Incidence rate per 100,000 person-day.

^b^Incidence rate ratios for outcomes with zero events were not estimated.

^c^Adjusted for age, sex, number of days from the index date to the local Omicron outbreak (January 1, 2022), clinical history of chronic conditions before the index date (Supplementary Table 5), and medications prescribed within 90 days before the index date (Supplementary Table 6).

## Discussion

In this study, we found that individuals with a previous SARS-CoV-2 infection and received one priming dose of either BNT162b2 or CoronaVac vaccine had a lower risk of a subsequent infection during the Omicron outbreak compared with other regular vaccine recipients without previous infection after their second dose. The incidence rates of COVID-19-related hospitalization and mortality were low in both groups, and we did not find any differential risk of COVID-19-related hospitalization and mortality between the two groups. The results are reassuring to people in Hong Kong and other regions with similar vaccination recommendation who received only one priming dose due to previous infection. Our findings suggest that hybrid immunity induced by the combination of infection and vaccination provide stronger protection against subsequent infection and similar protection against COVID-19-related hospitalization and mortality as the immunity induced by two priming doses of the COVID-19 vaccine amid the Omicron outbreak.

The finding is consistent with several studies suggesting that the antibody responses elicited by two doses of the mRNA vaccine are very similar to that elicited by a SARS-CoV-2 infection plus one dose of the vaccine^24,25^. A cohort study in Israel similarly reported that people who received a single dose of the vaccine after previous infection had lower risk of infection compared with two dose recipients without any previous infection^26^. Existing data mostly focused on the antibody levels rather than COVID-19-related infection, hospitalization, and mortality. We were only able to identify several studies investigating the risk of SARS-CoV-2 infection and COVID-19-related hospitalization among individuals with hybrid immunity. A cohort study in Sweden compared previously infected individuals who received COVID-19 vaccine with those who did not receive any COVID-19 vaccine after infection and found that both one-dose and two-dose immunity were associated with a lower risk of COVID-19 hospitalization than natural immunity alone^27^. Another cohort study in Qatar reported that individuals with a previous SARS-CoV-2 infection and received two doses of the mRNA vaccines had a lower risk of reinfection compared to fully vaccinated individuals without history of infection^28^. However, it is well recognized that both SARS-CoV-2 infection and COVID-19 vaccines can induce immunity and a higher level of immunity tends to provide better protection.

The humoral response elicited by post-infection vaccination might explain the lower risk of reinfection. A previous study on humoral immune responses found that vaccination after infection elicited a greater humoral response compared with vaccine or infection alone, with a longer time between infection and vaccination associated with an increasing post-vaccine anti-spike-immunoglobulin G peak^29^. Similar results were observed in several studies of different ethnicities where higher SARS-CoV-2 antibody levels in previously infected individuals after one dose of COVID-19 vaccine compared with infection-naive individuals after two doses^30–32^. A recent review comparing the efficacy and duration of natural and hybrid immunity also suggested that hybrid immunity appeared to confer the greater protection against SARS-CoV-2 infections, although there were still knowledge gaps on the underlying mechanism^14^. When we compared individuals who were previously infected but did not receive vaccination with fully vaccinated individuals without prior infection on the risk of COVID-19-related hospitalization, although the crude estimates suggested a significant lower risk in the infection group, the no significant difference between the two groups was detected after adjusting for potential confounders. We believe the difference in time since the last immune conferring event between groups contributed to the changes after adjustment. The group with a history of infection but unvaccinated were likely infected at a closer time before the Omicron outbreak and they were not yet eligible to receive a vaccine dose after recovery, while numerous vaccine recipients took their second dose early in time and the protection waned over time. Furthermore, the lower risk of infection remained among individuals with a previous SARS-CoV-2 infection and unvaccinated. This finding is consistent with a systematic review which revealed that the natural immunity in individuals recovered from COVID-19 was at least equivalent to the protection provided by complete vaccination in infection-naïve populations^33^. Among individuals who were previously infected with SARS-CoV-2, a population-based cohort study in Iceland identified a higher risk of reinfection in those who received two or more doses compared to one dose or fewer of vaccine, and the risk of reinfection increased with time from the initial infection, suggesting a differential level of protection against reinfection conferred by natural and hybrid immunity^34^. The natural immunity induced by a previous SARS-CoV-2 infection might generate a more extensive immune response to the SARS-CoV-2 proteins compared to that generated by the anti-spike protein immune activation induced by the COVID-19 vaccines^35–37^. However, the neutralizing antibody response in individuals recovered from COVID-19 varied largely^38^, and the role of different types of cellular immune remains unclear, which requires further investigations^39^. This finding may also reflect a ‘healthy survivor effect’ whereby individuals who survived a previous infection are likely to be healthier and therefore less likely to be reinfected. It is also possible that individuals with a previous infection are less likely to report their reinfection to the Department of Health especially when the symptoms are minor and do not require any medical treatment in the hospital. Additionally, previous SARS-CoV-2 infection may trigger behavioral changes including better personal hygiene practice, which reduces the risk of reinfection.

While most existing studies on vaccine effectiveness against SARS-CoV-2 infection excluded individuals with a previous SARS-CoV-2 infection^40–42^, this study compared the risk of COVID-19-related infection, hospitalization, and mortality during an Omicron outbreak between those with a previous SARS-CoV-2 infection and received one priming dose and fully vaccinated individuals with BNT162b2 or CoronaVac. The use of population-based vaccination records and territory-wide public healthcare database ensures the population representativeness of the study cohort. A series of sensitivity analysis demonstrate the robustness of the results. The self-reported RAT positive cases obtained from the Department of Health were included as a sensitivity analysis. The Hong Kong government requires all RAT positive results to be reported within one day after the testing date through an online declaration system. Individuals who test positive using RAT are encouraged to report their results to receive more appropriate support from the health authority. The infection reported through the system affects the eligibility for vaccination and getting a Vaccines Pass that allows individuals to enter public places such as restaurants and sports facilities. Individuals fail to report their positive results may also face legal liabilities. Under these public health policies, the data from the Department of Health should covered most infection cases using self-administered RAT. Despite these strengths, this study has several limitations. First, we only used test results provided by the Department of Health to define SARS-CoV-2 infection. Despite better specificity, we may have omitted infection cases determined only by self-testing kits and with no record in the Department of Health. As the number of undetected and underreported cases were high in many countries and regions^43^, this issue might occur in similar studies and lead to an underestimation of the protection against subsequent infection conferred by natural or hybrid immunity. Nevertheless, in Hong Kong, these cases were mostly asymptomatic or minor cases that do not require any treatment in the hospital, so it should not affect our estimates on the risk of COVID-19-related hospitalization and mortality. Second, the low incidence rates and the relatively small sample size in the group with previous infection limited the numbers of events we could observe within the study period, resulting in wide confidence intervals in some estimates, especially for the mortality outcome. The small event number also limited the feasibility to further examine the time-varying effectiveness of the hybrid immunity as well as the effectiveness of the booster doses. Although we were unable to examine the waning of immunity over time, the time from the last immune conferring event to the index date was similar across different groups, and we did include it as a covariate in the models to control for the potential confounding effect. Third, several studies have shown that the vaccine-induced immunity appeared to wane faster than that induced by a previous infection^14^, but we were not able to control for this in the analysis. Fourth, the study outcome may also vary across different variants of the previous SARS-CoV-2 infection, but we were unable to ascertain the variant of the previous SARS-CoV-2 infection due to limited data availability. Fifth, we considered all hospitalization with positive PCR test within 28 days before admission as COVID-19 hospitalization. This might include hospitalization due to non-COVID-19-related conditions but with positive PCR result, which might not indicate severe COVID-19 cases. Finally, this study is observational in nature and the results are subject to bias introduced by unmeasured confounders such as behavioral factors. Future studies could incorporate a more comprehensive list of potential confounders and further examine the effectiveness of the subsequent booster dose among individuals with a previous SARS-CoV-2 infection.

As the number of SARS-CoV-2 infections is still increasing worldwide, a sizeable fraction of the population needs to consider their history of infection in their subsequent vaccine uptake decisions, i.e., booster doses. Our data is apparently more in favor of clinical recommendations that patients who recovered from COVID-19 could receive one fewer dose of the vaccine to gain at least similar protection with people without an infection history. If substantiated by further research data and evidence, the government could consider an infection as a substitute for one dose of COVID-19 vaccines and prioritize vaccination for people without any previous infection, especially for countries and regions where vaccine supply is a concern. As hybrid immunity also tend to decline over time^26,44^, future research could investigate whether the second dose for individuals with previous infection receiving one priming dose could be considered as the booster dose to achieve a similar level of protection as the regular vaccination regimen for individuals with no previous infection. Since we were only able to examine hybrid immunity within several months given the available data, the results might not be generalizable to time beyond our observation period. To our knowledge, there is only one study investigating the time-varying effects of vaccine-induced and hybrid immunity with a relatively small sample size^45^. Future studies should also examine the duration of hybrid immunity at a population level and develop an effective vaccination schedule for individuals with a previous SARS-CoV-2 infection to achieve an optimal level of protection over time.

In conclusion, the hybrid immunity from single dose vaccine and previous variants of SARS-CoV-2 conferred a stronger level of protection against Omicron infection and a similar level of protection against hospitalization and mortality, compared with the immunity induced by two priming doses of the vaccine without previous infection. Our findings support clinical recommendations that individuals with a previous SARS-CoV-2 infection could receive a single dose to gain at least similar protection as those who received two doses without previous infection.

## Methods

### Data source

We obtained population-based vaccination record and positive SARS‑CoV‑2 PCR results from the Department of Health. Territory-wide de-identified electronic medical records (EMRs) between January 1, 2018 and March 31, 2022 were provided by the Hospital Authority (HA), the statutory body managing all public hospital services in Hong Kong. We performed data linkage between vaccination records, positive PCR results, and EMRs by matching unique pseudo-ID to protect patient privacy. Previous COVID-19 vaccine pharmacovigilance studies have been conducted using this database^46–48^. This study was approved by the Hospital Authority Central Institutional Review Board (CIRB-2021-005-4) and the Department of Health Ethics Committee (LM171/2021).

### Study design

This was a retrospective cohort study with the analysis stratified by vaccine type, i.e., BNT162b2 and CoronaVac. The study cohort consisted of all individuals aged 12 years or above and vaccinated before January 1, 2022, the month when the first local Omicron BA.2 case was identified, with either one dose of COVID-19 vaccine after a SARS‑CoV‑2 infection or with two doses of COVID-19 vaccines and with no history of infection upon receiving their second dose. Individuals with a previous SARS‑CoV‑2 infection and received two vaccines doses before the Omicron outbreak were not included in the study cohort. Those who received one dose of the vaccine and then got infected before the Omicron outbreak were also excluded from the study cohort. For individuals with a history of SARS‑CoV‑2 infection and received one dose of the vaccine, the index date was the date of taking the first dose of either BNT162b2 or CoronaVac. For individuals with no prior infection, the index date was the date of receiving the second dose of either BNT162b2 or CoronaVac. The observation started from the index date and ended upon outcome occurrence, death, receiving an additional dose of the COVID-19 vaccine, or the end date of data availability (March 31, 2022), whichever came the earliest. Considering the waning of protection conferred by the latest dose of the vaccines, the time from the index date to January 1, 2022 was included for multivariable adjustment (please see details in “Statistical analysis”).

### Exposure

The exposure of the study was any SARS-CoV-2 infection defined by PCR-positive result before receiving the first dose of COVID-19 vaccine. The exposed group consisted of individuals who received their first dose of BNT162b2 or CoronaVac after a SARS-CoV-2 infection. The unexposed group included individuals who received two doses of BNT162b2 or CoronaVac and with no prior SARS-CoV-2 infection.

### Outcomes

The outcomes of this study were SARS-CoV-2 infection, COVID-19-related hospitalization, and COVID-19-related mortality during the Omicron outbreak in Hong Kong starting from January 1, 2022. SARS-CoV-2 infection was defined by PCR-positive result. COVID-19-related hospitalization and mortality were defined by a PCR positive test result within 28 days before admission or death.

### Statistical analysis

We stratified the cohort by vaccine type. Descriptive statistics were used to characterize the study cohort. Poisson regression models were used to calculate the IRR of SARS‑CoV‑2 infection, COVID-19-related hospitalization, and COVID-19-related mortality between vaccine recipients with and without prior SARS-CoV-2 infection with the follow up period (in person-day) as the offset term. In an extended analysis, we also compared those who had a previous infection without vaccination (index date being the date of positive PCR test result) and those who received the regular two-dose regimen with no prior infection to compare the protection conferred by natural immunity and vaccine-induced immunity. Some evidence show that natural immunity offers equal or greater protection against SARS-CoV-2 infections compared to immunity induced by two doses of the COVID-19 vaccines, but the existing data are not consistent^14^. We hypothesized that a previous SARS-CoV-2 infection might provide similar protection against infection, hospitalization, and death as the two-dose vaccination regimen. We included the following covariates in the multivariable models for possible confounding effects: age, sex, number of days from the index date to the local Omicron outbreak (January 1, 2022), clinical history of chronic conditions before the index date (Supplementary Table 5), and medications prescribed within 90 days before the index date (Supplementary Table 6).

A series of sensitivity analysis were conducted to examine the robustness of the results. First, we changed the time window defining COVID-19-related outcome events from 28 days to 14 and 42 days, respectively, to cover fewer or more possible events. Second, as evidence showed that the vaccine recipients usually reached the optimal protection level about 2 weeks after being fully vaccinated^49^, we postponed the index date by 14 days to better compare the optimal protection effects between the exposed and unexposed groups. Third, we included self-reported cases using RAT to define outcome events as these cases, although may not be confirmed by PCR, accounted for a considerable proportion of the total infection cases in the local Omicron epidemic. All analyses were conducted in R statistical environment (Version 4.2.1, Vienna, Austria). L.H. and V.K.C.Y. independently conducted the analyses and cross-checked to ensure the accuracy of the results.

### Reporting summary

Further information on research design is available in the Nature Research Reporting Summary linked to this article.

## Supplementary information

Supplementary information

REPORTING SUMMARY

## Data Availability

Data will not be available for others as the data custodians have not given permission.

## Code availability

All code for data analysis associated with the current study is available upon request.
