# Supplementary material for: Comparing hybrid and regular COVID-19 vaccine-induced immunity against the Omicron epidemic
Source: NPJ Vaccines. 2022 Dec 15;7:162. doi: 10.1038/s41541-022-00594-7 (PMC9753877; doi:10.1038/s41541-022-00594-7)
Supplement: Supplementary file 1 — Supplementary information [file 41541_2022_594_MOESM1_ESM.pdf]

Supplementary Table 1 Sensitivity analysis with outcomes defined as PCR test positive within 14 days before hospitalization or mortality

COVID-19 hospitalization	Number of individuals	Event	Total duration of follow-up (person-days)	Incidence rate[1]	Incidence rate ratio (95% confidence interval)[2], *p*-value	
					Crude	Adjusted[3]
BNT162b2						
Without prior SARS-CoV-2 infection	1,556,405	2,871	344,739,111	0.833	Ref	Ref
With prior SARS-CoV-2 infection	5,389	4	1,162,207	0.344	0.413 (0.156, 1.098), 0.076	0.450 (0.169, 1.195), 0.109
CoronaVac						
Without prior SARS-CoV-2 infection	1,105,711	2,609	233,891,272	1.115	Ref	Ref
With prior SARS-CoV-2 infection	2,026	2	385,545	0.519	0.465 (0.116, 1.857), 0.279	0.504 (0.126, 2.018), 0.333
COVID-19 mortality						
BNT162b2						
Without prior SARS-CoV-2 infection	1,556,405	78	344,887,368	0.023	Ref	Ref
With prior SARS-CoV-2 infection	5,389	0	1,162,367	0.000	-	-
CoronaVac						
Without prior SARS-CoV-2 infection	1,105,711	272	233,980,242	0.116	Ref	Ref
With prior SARS-CoV-2 infection	2,026	0	385,579	0.000	-	-

[1] Incidence rate per 100,000 person-day [2] Incidence rate ratios for outcomes with zero events were not estimated [3] Adjusted for age, sex, number of days from the index date to the local Omicron outbreak (January 1, 2022), clinical history of chronic conditions before the index date (Supplementary Table 5), and medications prescribed within 90 days before the index date (Supplementary Table 6).

Supplementary Table 2 Sensitivity analysis with outcomes defined as PCR test positive within 42 days before mortality or hospitalization

COVID-19 hospitalization	Number of individuals	Event	Total duration of follow-up (person-days)	Incidence rate[1]	Incidence rate ratio (95% confidence interval)[2], *p*-value	
					Crude	Adjusted[3]
BNT162b2						
Without prior SARS-CoV-2 infection	1,556,405	3,454	344,733,814	1.002	Ref	Ref
With prior SARS-CoV-2 infection	5,389	4	1,162,207	0.344	0.344 (0.129, 0.913), 0.032	0.383 (0.144, 1.017), 0.054
CoronaVac						
Without prior SARS-CoV-2 infection	1,105,711	3,123	233,886,871	1.335	Ref	Ref
With prior SARS-CoV-2 infection	2,026	2	385,545	0.519	0.388 (0.097, 1.551), 0.181	0.420 (0.105, 1.682), 0.221
COVID-19 mortality						
BNT162b2						
Without prior SARS-CoV-2 infection	1,556,405	99	344,887,368	0.029	Ref	Ref
With prior SARS-CoV-2 infection	5,389	0	1,162,367	0.000	-	-
CoronaVac						
Without prior SARS-CoV-2 infection	1,105,711	357	233,980,242	0.153	Ref	Ref
With prior SARS-CoV-2 infection	2,026	0	385,579	0.000	-	-

[1] Incidence rate per 100,000 person-day [2] Incidence rate ratios for outcomes with zero events were not estimated [3] Adjusted for age, sex, number of days from the index date to the local Omicron outbreak (January 1, 2022), clinical history of chronic conditions before the index date (Supplementary Table 5), and medications prescribed within 90 days before the index date (Supplementary Table 6).

Supplementary Table 3 Sensitivity analysis with index date postponed by 14 days

SARS-CoV-2 infection	Number of individuals	Event	Total duration of follow-up (person-days)	Incidence rate[1]	Incidence rate ratio (95% confidence interval)[2], *p*-value	
					Crude	Adjusted[3]
BNT162b2						
Without prior SARS-CoV-2 infection	1,556,405	126,906	319,305,217	39.744	Ref	Ref
With prior SARS-CoV-2 infection	5,389	178	1,081,764	16.455	0.414 (0.357, 0.480), <0.001	0.477 (0.411, 0.552), <0.001
CoronaVac						
Without prior SARS-CoV-2 infection	1,105,711	92,899	215,861,744	43.036	Ref	Ref
With prior SARS-CoV-2 infection	2,026	61	355,499	17.159	0.399 (0.310, 0.512), <0.001	0.399 (0.310, 0.513), <0.001
COVID-19 hospitalization						
BNT162b2						
Without prior SARS-CoV-2 infection	1,556,405	3,342	322,944,958	1.035	Ref	Ref
With prior SARS-CoV-2 infection	5,389	4	1,086,761	0.368	0.356 (0.134, 0.944), 0.038	0.396 (0.149, 1.051), 0.063
CoronaVac						
Without prior SARS-CoV-2 infection	1,105,711	3,026	218,407,280	1.385	Ref	Ref
With prior SARS-CoV-2 infection	2,026	2	357,181	0.560	0.404 (0.101, 1.613), 0.199	0.435 (0.109, 1.740), 0.239
COVID-19 mortality						
BNT162b2						
Without prior SARS-CoV-2 infection	1,556,405	94	323,097,698	0.029	Ref	Ref
With prior SARS-CoV-2 infection	5,389	0	1,086,921	0.000	-	-
CoronaVac						
Without prior SARS-CoV-2 infection	1,105,711	350	218,500,288	0.160	Ref	Ref
With prior SARS-CoV-2 infection	2,026	0	357,215	0.000	-	-

[1] Incidence rate per 100,000 person-day [2] Incidence rate ratios for outcomes with zero events were not estimated [3] Adjusted for age, sex, number of days from the index date to the local Omicron outbreak (January 1, 2022), clinical history of chronic conditions before the index date (Supplementary Table 5), and medications prescribed within 90 days before the index date (Supplementary Table 6).

Supplementary Table 4 Sensitivity analysis including self-reported infection cases using rapid antigen tests

SARS-CoV-2 infection	Number of individuals	Event	Total duration of follow-up (person-days)	Incidence rate[1]	Incidence rate ratio (95% confidence interval)[2], *p*-value	
					Crude	Adjusted[3]
BNT162b2						
Without prior SARS-CoV-2 infection	1,556,405	199,570	339,459,249	58.791	Ref	Ref
With prior SARS-CoV-2 infection	5,389	242	1,155,824	20.937	0.356 (0.314, 0.404), <0.001	0.415 (0.366, 0.471), <0.001
CoronaVac						
Without prior SARS-CoV-2 infection	1,105,711	123,686	230,642,630	53.627	Ref	Ref
With prior SARS-CoV-2 infection	2,026	84	383,388	21.910	0.409 (0.330, 0.506), <0.001	0.410 (0.331, 0.508), <0.001
COVID-19 hospitalization						
BNT162b2						
Without prior SARS-CoV-2 infection	1,556,405	3,589	344,731,926	1.041	Ref	Ref
With prior SARS-CoV-2 infection	5,389	4	1,162,207	0.344	0.331 (0.124, 0.878), 0.026	0.371 (0.140, 0.985), 0.047
CoronaVac						
Without prior SARS-CoV-2 infection	1,105,711	3,245	233,885,126	1.387	Ref	Ref
With prior SARS-CoV-2 infection	2,026	2	385,545	0.519	0.374 (0.094, 1.493), 0.164	0.405 (0.101, 1.619), 0.201
COVID-19 mortality						
BNT162b2						
Without prior SARS-CoV-2 infection	1,556,405	97	344,887,368	0.028	Ref	Ref
With prior SARS-CoV-2 infection	5,389	0	1,162,367	0.000	-	-
CoronaVac						
Without prior SARS-CoV-2 infection	1,105,711	368	233,980,242	0.157	Ref	Ref
With prior SARS-CoV-2 infection	2,026	0	385,579	0.000	-	-

[1] Incidence rate per 100,000 person-day [2] Incidence rate ratios for outcomes with zero events were not estimated [3] Adjusted for age, sex, number of days from the index date to the local Omicron outbreak (January 1, 2022), clinical history of chronic conditions before the index date (Supplementary Table 5), and medications prescribed within 90 days before the index date (Supplementary Table 6).

Supplementary Table 5 Diagnostic codes used to operationalize chronic conditions

Chronic conditions	International Classification of Diseases, Ninth Revision	International Classification of Primary Care
Alcohol misuse	265.2, 291.1–291.3, 291.5–291.9, 303.0, 303.9, 305.0, 357.5, 425.5, 535.3, 571.0–571.3, 980, V11.3	P15
Asthma	493	R96
Atrial fibrillation	427.3	K78
Cancer, lymphoma	200–202, 203.0, 238.6	B72
Cancer, metastatic	196–199	B74, D74, D76, D77, L71, N74, S77, T71, U75, U76, U77, W72, X77, Y78
Cancer, non-metastatic	153-154, 162-163, 174, 180, 185, 230.3-230.6, 231.2, 233.0-233.1, 233.4	D75, R84, X75, X76, Y77
Chronic heart failure	398.91, 402.01, 402.11, 402.91, 404.01, 404.03, 404.11, 404.13, 404.91, 404.93, 425.4–425.9, 428	K77
Chronic kidney disease	583, 584, 585, 586, 592, 593.9	U14
Chronic pain	307.80, 307.89, 338.0, 338.2, 338.4, 719.41, 719.45 - 719.47, 719.49, 720.0, 720.2, 720.9, 721.0 - 721.4, 721.6, 721.8, 721.9, 722, 723.0, 723.1, 723.3 - 723.9, 724.0 - 724.6, 724.70, 724.79, 724.8, 724.9, 729.0 - 729.2, 729.4, 729.5	A01
Chronic pulmonary disease	416.8, 416.9, 490–492, 494-505, 506.4, 508.1, 508.8	R95
Chronic viral hepatitis B	70.2-70.3	D72
Cirrhosis	571.2, 571.5, 571.6, 456.0, 456.1, 456.20, 456.21, 567.0, 567.2, 567.21, 567.29, 567.8, 567.9, 572.2, 572.4, 789.5	-
Dementia	290, 294.1, 331.2	P70
Depression	296.2, 296.3, 296.5, 300.4, 309, 311	P76
Diabetes	250	T89-T90
Epilepsy	345	N88
Hypertension	401-405	K86-K87
Hypothyroidism	240.9, 243, 244, 246.1, 246.8	T86
Inflammatory bowel disease	555, 556	-
Irritable bowel syndrome	564.1	D93
Multiple sclerosis	323, 340, 341.0, 341.9, 377.3	N86
Myocardial infarction	410	K75
Parkinson’s disease	332	N87
Peptic ulcer disease	531.7, 531.9, 532.7, 532.9, 533.7, 533.9, 534.7, 534.9	D86
Peripheral vascular disease	440.2	K92
Psoriasis	696.1	S91
Rheumatoid arthritis	446.5, 710.0–710.4, 714.0–714.2, 714.8, 725	L88
Schizophrenia	295	P72
Severe constipation	560.1, 560.30, 560.39, 560.9, 564.0, 569.83, 569.89	D12
Stroke or transient ischemic attack	362.3, 430, 431, 433.x1, 434.x1, 435, 436	K90

Supplementary Table 6 British National Formulary codes for the operationalization of medication use

Medications	British National Formulary code
Renin-angiotensin-system agents	2.5.5
Beta blockers	2.4
Calcium channel blockers	2.6.2
Diuretics	2.2
Nitrates	2.6.1
Lipid lowering agents	2.12
Insulins	6.1.1
Antidiabetic drugs	6.1.2
Antiarrhythmic drugs	2.3.2
Oral anticoagulants	2.8.2
Antiplatelets	2.9
Steroid	6.3.2
Antidepressants	4.3
Antiviral drugs	5.3
Antibacterial drugs	5.1
Immunosuppressants	8.2
