# Supplementary material for: Comparing hybrid and regular COVID-19 vaccine-induced immunity against the Omicron epidemic
Source: NPJ Vaccines. 2022 Dec 15;7:162. doi: 10.1038/s41541-022-00594-7 (PMC9753877; doi:10.1038/s41541-022-00594-7)
Supplement: Supplementary file 2 — REPORTING SUMMARY [file 41541_2022_594_MOESM2_ESM.pdf]

nature portfolio

Corresponding author(s): Esther Wai Yin Chan

Last updated by author(s): Oct 12, 2022

Reporting Summary

Nature Portfolio wishes to improve the reproducibility of the work that we publish. This form provides structure for consistency and transparency in reporting. For further information on Nature Portfolio policies, see our Editorial Policies and the Editorial Policy Checklist.

Statistics

For all statistical analyses, confirm that the following items are present in the figure legend, table legend, main text, or Methods section.

n/a	Confirmed	
☐	☒	The exact sample size (*n*) for each experimental group/condition, given as a discrete number and unit of measurement
☒	☐	A statement on whether measurements were taken from distinct samples or whether the same sample was measured repeatedly
☐	☒	The statistical test(s) used AND whether they are one- or two-sided<br>*Only common tests should be described solely by name; describe more complex techniques in the Methods section.*
☐	☒	A description of all covariates tested
☐	☒	A description of any assumptions or corrections, such as tests of normality and adjustment for multiple comparisons
☐	☒	A full description of the statistical parameters including central tendency (e.g. means) or other basic estimates (e.g. regression coefficient) AND variation (e.g. standard deviation) or associated estimates of uncertainty (e.g. confidence intervals)
☐	☒	For null hypothesis testing, the test statistic (e.g. *F*, *t*, *r*) with confidence intervals, effect sizes, degrees of freedom and *P* value noted<br>*Give P values as exact values whenever suitable.*
☒	☐	For Bayesian analysis, information on the choice of priors and Markov chain Monte Carlo settings
☐	☒	For hierarchical and complex designs, identification of the appropriate level for tests and full reporting of outcomes
☒	☐	Estimates of effect sizes (e.g. Cohen's *d*, Pearson's *r*), indicating how they were calculated

Our web collection on statistics for biologists contains articles on many of the points above.

Software and code

Policy information about availability of computer code

Data collection	No specific software was used in the collection of data.
Data analysis	All analyses were conducted using the R statistical environment (Version 4.2.1, Vienna, Austria).

For manuscripts utilizing custom algorithms or software that are central to the research but not yet described in published literature, software must be made available to editors and reviewers. We strongly encourage code deposition in a community repository (e.g. GitHub). See the Nature Portfolio guidelines for submitting code & software for further information.

Data

Policy information about availability of data

All manuscripts must include a data availability statement. This statement should provide the following information, where applicable:

- Accession codes, unique identifiers, or web links for publicly available datasets
- A description of any restrictions on data availability
- For clinical datasets or third party data, please ensure that the statement adheres to our policy

Data will not be available for others as the data custodians have not given permission.

Human research participants

Policy information about studies involving human research participants and Sex and Gender in Research.

Reporting on sex and gender	Data on sex were obtained from medical records. Sex was included as one of the covariates in the analysis and descriptive statistics were reported in the results section. Among BNT162b2 recipients, the proportion of males was higher in the group with prior infection (49.1%) than that in group without prior infection (44.9%), and the mean age (in year) was younger in the group without prior infection (Mean = 45.85, Standard deviation [SD] = 17.04) compared to the group with prior infection (Mean = 44.28, SD = 16.40). Among CoronaVac recipients, sex was similar between the two groups while the mean age was younger in the group without prior infection (Mean = 56.86, SD = 14.40) compared to the group with prior infection (Mean = 54.93, SD = 14.56). This study used secondary data and no individual-level consent was required.
Population characteristics	We included the following covariates in the multivariable models for possible confounding effects: age, sex, number of days from the index date to the local Omicron outbreak (January 1, 2022), clinical history of chronic conditions before the index date (Supplementary Table 5), and medications prescribed within 90 days before the index date (Supplementary Table 6).
Recruitment	This study used secondary data and no recruitment is involved.
Ethics oversight	This study was approved by the Hospital Authority Central Institutional Review Board (CIRB-2021-005-4) and the Department of Health Ethics Committee (LM171/2021).

Note that full information on the approval of the study protocol must also be provided in the manuscript.

Field-specific reporting

Please select the one below that is the best fit for your research. If you are not sure, read the appropriate sections before making your selection.

☒ Life sciences ☐ Behavioural & social sciences ☐ Ecological, evolutionary & environmental sciences

For a reference copy of the document with all sections, see nature.com/documents/nr-reporting-summary-flat.pdf

Life sciences study design

All studies must disclose on these points even when the disclosure is negative.

Sample size	No sample size calculation was conducted because of the novel use of COVID-19 vaccines, it was not possible for this pharmacovigilance to predict the effect size. Methodologically, this is a population-based study and all identified eligible patients from the territory-wide healthcare database were included in the analysis.
Data exclusions	Individuals with a previous SARS□CoV□2 infection and received two vaccines doses before the Omicron outbreak were not included in the study cohort. Those who received one dose of the vaccine and then got infected before the Omicron outbreak were also excluded from the study cohort.
Replication	A series of sensitivity analyses were conducted on the same study sample, with no substantial deviation from the main results observed.
Randomization	This is an observational study.
Blinding	This is an observational study.

Reporting for specific materials, systems and methods

We require information from authors about some types of materials, experimental systems and methods used in many studies. Here, indicate whether each material, system or method listed is relevant to your study. If you are not sure if a list item applies to your research, read the appropriate section before selecting a response.

Materials & experimental systems

n/a	Involved in the study	
☒	☐	Antibodies
☒	☐	Eukaryotic cell lines
☒	☐	Palaeontology and archaeology
☒	☐	Animals and other organisms
☐	☒	Clinical data
☒	☐	Dual use research of concern

Methods

n/a	Involved in the study	
☒	☐	ChIP-seq
☒	☐	Flow cytometry
☒	☐	MRI-based neuroimaging

Clinical data

Policy information about clinical studies

All manuscripts should comply with the ICMJE guidelines for publication of clinical research and a completed CONSORT checklist must be included with all submissions.

Clinical trial registration	This is not a clinical trial
Study protocol	There is no openly available study protocol
Data collection	We obtained population-based vaccination record and positive SARS?CoV?2 PCR results from the Department of Health. Territory-wide de-identified electronic medical records (EMRs) between January 1, 2018, and March 31, 2022, were provided by the Hospital Authority (HA), the statutory body managing all public hospital services in Hong Kong.
Outcomes	The outcomes of this study were SARS-CoV-2 infection, COVID-19-related hospitalization, and COVID-19-related mortality during the Omicron outbreak in Hong Kong starting from January 1, 2022. SARS-CoV-2 infection was defined by PCR-positive result. COVID-19-related hospitalization and mortality were defined by a PCR positive test result within 28 days before admission or death.
